# A tale of two technologies: Use of cardiac contractility modulator with wearable cardiac defibrillator

**DOI:** 10.1016/j.hrcr.2023.10.019

**Published:** 2023-10-31

**Authors:** Marissa Heyer, Anish Trivedi, Matthew Fata, Kanika Mody, Sameer M. Jamal

**Affiliations:** ∗ Department of Internal Medicine, Hackensack University Medical Center, Hackensack, New Jersey; † New York Institute of Technology – Long Island Campus, Old Westbury, New York; ‡ Department of Internal Medicine, Division of Cardiology, Hackensack University Medical Center, Hackensack, New Jersey; § Heart Failure and Mechanical Circulatory Support, Hackensack University Medical Center, Hackensack, New Jersey; ‖ Division of Cardiac Electrophysiology, Department of Cardiology, Hackensack University Medical Center, Hackensack, New Jersey; ¶ Hackensack Meridian School of Medicine, Nutley, New Jersey

**Keywords:** Cardiac contractility modulation, Wearable cardiac defibrillator, Cross-device interaction, Heart failure, Ventricular arrhythmia

**Key Teaching Points**

• It is important for healthcare providers to consider the potential risk of cross-device interactions in patients who require multiple devices—particularly as new devices and technology continue to emerge.
• Cardiac contractility modulation (CCM) therapy and the LifeVest wearable cardiac defibrillator can be safely used in combination, as no interaction between the 2 devices was observed during provocative testing. However, it is important to consider theoretical interactions between the 2 devices and explore specific algorithms programmed for each device to mitigate such potential risks.
• Research investigating CCM therapy during inotrope wean has been limited overall. Our case demonstrates a potential use of CCM therapy in those with recent or active milrinone use, as our patient demonstrated full recovery of left ventricular ejection fraction 5 months post device implantation.

## Introduction

Advancements in technology have provided a greater ability to treat cardiac conditions. Accordingly, as healthcare providers consider these options for their patients, there remains a potential risk for cross-device interactions in the event a patient requires multiple devices.

The wearable cardiac defibrillator (WCD) is a device specifically created to detect and halt ventricular arrhythmias. The LifeVest by Zoll Medical (Pittsburgh, PA) is an FDA-approved WCD indicated to reduce the risk of sudden cardiac death (SCD) in a vulnerable population.^1^

Cardiac contractility modulation (CCM) therapy optimizes cardiac muscle contraction in patients with heart failure with reduced and midrange left ventricular ejection fractions (LVEF) by delivering nonexcitatory electrical signals during the absolute refractory period.^2^ The Optimizer Smart by Impulse Dynamics (Marlton, NJ) is a CCM device approved by the FDA to improve 6-minute hall walk distance, quality of life, and NYHA class functional status in those with heart failure who are not candidates for cardiac resynchronization therapy (CRT).

Through embedded electrodes, the LifeVest constantly analyzes electrical signals, introducing a theoretical interaction with a CCM device. Patients with a concomitant cardiovascular implantable electronic device, including implantable cardioverter-defibrillators and pacemakers, require provocative testing of CCM therapy at the time of CCM implant for cardiovascular implantable electronic device oversensing of CCM signals.^3^^,^^4^ There is a paucity of data describing the interactions between the LifeVest and CCM devices.

We present a case of a patient with both CCM and LifeVest, which yielded no clinically significant interaction between the LifeVest and CCM devices during provocative testing as well as wear duration, suggesting that the 2 devices can be safely used in combination.

## Case report

A 71-year-old female patient with a history of coronary artery disease with prior percutaneous coronary intervention, hypertension, hyperlipidemia, and transient ischemic attack but previously preserved LVEF presented to the emergency department with chest pain, nausea, and vomiting. On initial assessment, she was normotensive (129/70 mm Hg) and afebrile (97.8°F; 36.6°C) with a heart rate of 95 beats per minute. Of note, she was requiring supplemental oxygen via nasal cannula with saturations at 95%, which was new from her baseline. Physical examination revealed an ill-appearing female patient in moderate distress with dry mucosa. She was in respiratory distress with decreased breath sounds at the bilateral lung bases; cardiac exam revealed regular rate and rhythm with tenderness upon palpation of anterior chest wall but no appreciable murmurs. Additionally, she had 1+ pitting edema in the lower extremities bilaterally and decreased dorsalis pedis pulses.

Initial labs were notable for leukocytosis (white blood cell count 22.9 × 10^3^/μL) and thrombocytosis (498 × 10^3^/μL); hemoglobin and hematocrit were stable from her baseline (12.2 g/dL and 40.3%, respectively). Troponin-I was mildly elevated at 0.21 ng/mL with a brain natriuretic peptide of 603 pg/mL and lactate of 4.0 mmol/L. Additionally, the patient was hyponatremic and hyperkalemic (Na 131 mmol/L, K 5.3 mmol/L) with evidence of mild acute kidney injury (Cr 1.13 mg/dL with baseline approximately 0.8 mg/dL). She was also found to have diabetic ketoacidosis with blood glucose 598 mg/dL, as well as elevated anion gap (22) and beta-hydroxybutyrate (6.4 mmol/L).

Electrocardiogram revealed sinus tachycardia with new nonspecific T-wave abnormalities; she was also found to have a rising troponin-I to peak at 53.0 ng/mL, consistent with non–ST segment elevation myocardial infarction. Left heart catheterization demonstrated severe multivessel disease with heavily calcified, small-caliber vessels and LVEF of 20%. Additionally, right heart catheterization revealed a wedge pressure of 25 mm Hg, pulmonary artery saturation of 41.4%, and calculated cardiac output and cardiac index of 2.5 and 1.51, respectively. She underwent urgent coronary artery bypass graft (CABG) surgery × 2 using left internal mammary artery to left anterior descending artery and endoscopically harvested left inferior saphenous vein to obtuse marginal artery with mitral valve annuloplasty.

**Figure 1:**
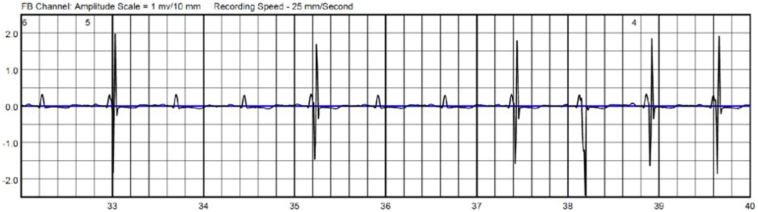
When cardiac contractility modulation therapy, as demonstrated by large-amplitude spikes following native QRS signals, is intermittent, the LifeVest device (Zoll Medical) may not be able to adjust gain appropriately, leading to potentially undersensing intrinsic QRS complexes.

Her hospital course was complicated by new-onset congestive heart failure initially requiring inotropes, with hypotension limiting use of guideline-directed medical therapy. An echocardiogram 3 weeks after CABG revealed LVEF 30%. A decision was made to implant a CCM device with inotrope wean, accepting the limited data in this space. The CCM device was implanted without incident (Figure 1) and she was successfully weaned off intravenous inotropes. She was fitted with a LifeVest WCD and discharged to a comprehensive inpatient rehabilitation program.

Her LVEF remained severely reduced at 25%–30% by echocardiogram nearly 3 months after CABG and 6 weeks post CCM placement. Her functional status continued to improve with minimal to no symptoms upon exertion. Subsequent echocardiogram at 4 months post CCM demonstrated improvement in LVEF to 35%–40% with mild anteroseptal and apical akinesia and grade 1 diastolic dysfunction. No ventricular arrhythmia was noted on the LifeVest monitoring system. As she was no longer considered an implantable cardioverter-defibrillator candidate, the LifeVest was removed. Most recently, her LVEF has recovered to 55%–60% at 7 months post CCM insertion.

## Discussion

CCM therapy is recommended for patients with an LVEF between 25% and 45% and NYHA class III who are ineligible for CRT. Mechanistically, both CCM and milrinone lead to increased calcium availability in the myocyte sarcomere and improve contractility.^5^ Unlike inotropes, however, CCM therapy does not increase myocardial oxygen consumption or arrhythmia burden.^6^ Although NYHA class III and ambulatory class IV patients were included, clinical trials of CCM such as the FIX-HF 5C study excluded those with either acute or chronic use of inotropic support.^7^ We are unaware of previous reports describing the use of CCM in those with recent or active milrinone use. Our patient did well after CCM implantation and her LVEF recovered fully at least 5 months after CABG, suggesting a positive influence of CCM therapy on LVEF improvement.

**Figure 2:**
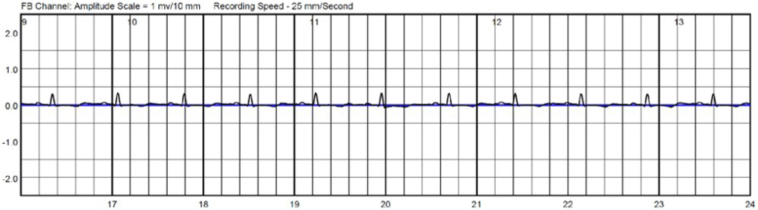
The gain of the LifeVest (Zoll Medical) gradually increases, increasing the sensitivity to detect smaller signals, in the absence of cardiac contractility modulation therapy.

**Figure 3:**
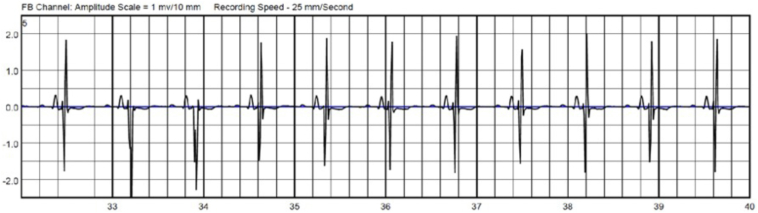
Cardiac contractility modulation therapy delivery time was extended maximally to 85 ms after QRS with no evidence of “double counting” or oversensing.

CCM devices contain built-in algorithms that inhibit therapy delivery with sensed rates above 110 beats per minute to reduce arrhythmia risk.^2^ Another important factor to consider is the potential effect of normal CCM therapy delivery on LifeVest arrhythmia detection. CCM delivers relatively high-energy output (7.5 V over 20 ms) during the absolute refractory period for several noncontiguous hours during a 24-hour period. The LifeVest adjusts internal signal amplification (gain) every 2 seconds, allowing for real-time, gradual adjustments to changes in cardiac signal voltage. The gain number provided on the example LifeVest tracings below are noted at the top of the tracing, and increases indicate more amplification while decreases indicate less (Figure 1, Figure 2, Figure 3).

To date, there has been little data analyzing interactions between CCM and WCDs.^3^ Although no interactions occurred in this case, we highlight a theoretical interaction between CCM therapy and LifeVest. The LifeVest could theoretically undersense QRS complexes during these 2-second periods when CCM therapy periodically turns off. For example, if CCM therapy delivery varies over several QRS complexes, as shown in Figure 1, the LifeVest may undersense native QRS complexes (undersensing confirmed using proprietary Zoll software by S. Syzmkiewicz, personal communication, June 17, 2022). It should be noted that the gain number in the tracing decreases as CCM is delivered after a greater number of QRS complexes.

The gain on the LifeVest increases in the absence of CCM therapy (Figure 2). If ventricular fibrillation, generally a low-amplitude rhythm, should occur during CCM therapy delivery, the CCM device would immediately withhold CCM therapy, as it is programmed to discontinue therapy for ventricular rates greater than 110 beats per minute. The LifeVest, however, could theoretically require 1 or more gain adjustments before appropriately detecting ventricular fibrillation.

CCM therapy is typically programmed to be delivered 35 ms after initial device QRS detection. We also provocatively extended CCM therapy delivery time to a maximal delay of 80 ms, as noted in Figure 3. The LifeVest did not oversense these therapies. There was no evidence of interference or oversensing between CCM therapy and LifeVest detection.

## Conclusion

This case report demonstrates 2 unique concepts. First, it highlights a potential use of CCM therapy during inotropic wean. Our patient had improvement of her LVEF after her surgery even while being titrated off of milrinone, which reflects a positive effect of CCM on LV recovery. Second, our case assessed potential sensing abnormalities between CCM therapy and LifeVest arrhythmia detection. Despite provocative evaluation, we did not identify any sensing abnormalities. Our case suggests that these 2 therapies can be simultaneously used in a safe manner. Additional reports and investigation are needed to draw more concrete conclusions.

## Disclosures

Education grant from Impulse Dynamics (Dr Jamal), Speakers bureau for Impulse Dynamics (Dr Mody). The remaining authors have no conflicts of interest to disclose.
